# Supplementary figures and images for: Rapid detection of myeloid neoplasm fusions using single-molecule long-read sequencing
Source: PLOS Glob Public Health. 2023 Sep 12;3(9):e0002267. doi: 10.1371/journal.pgph.0002267 (PMC10497132; doi:10.1371/journal.pgph.0002267)

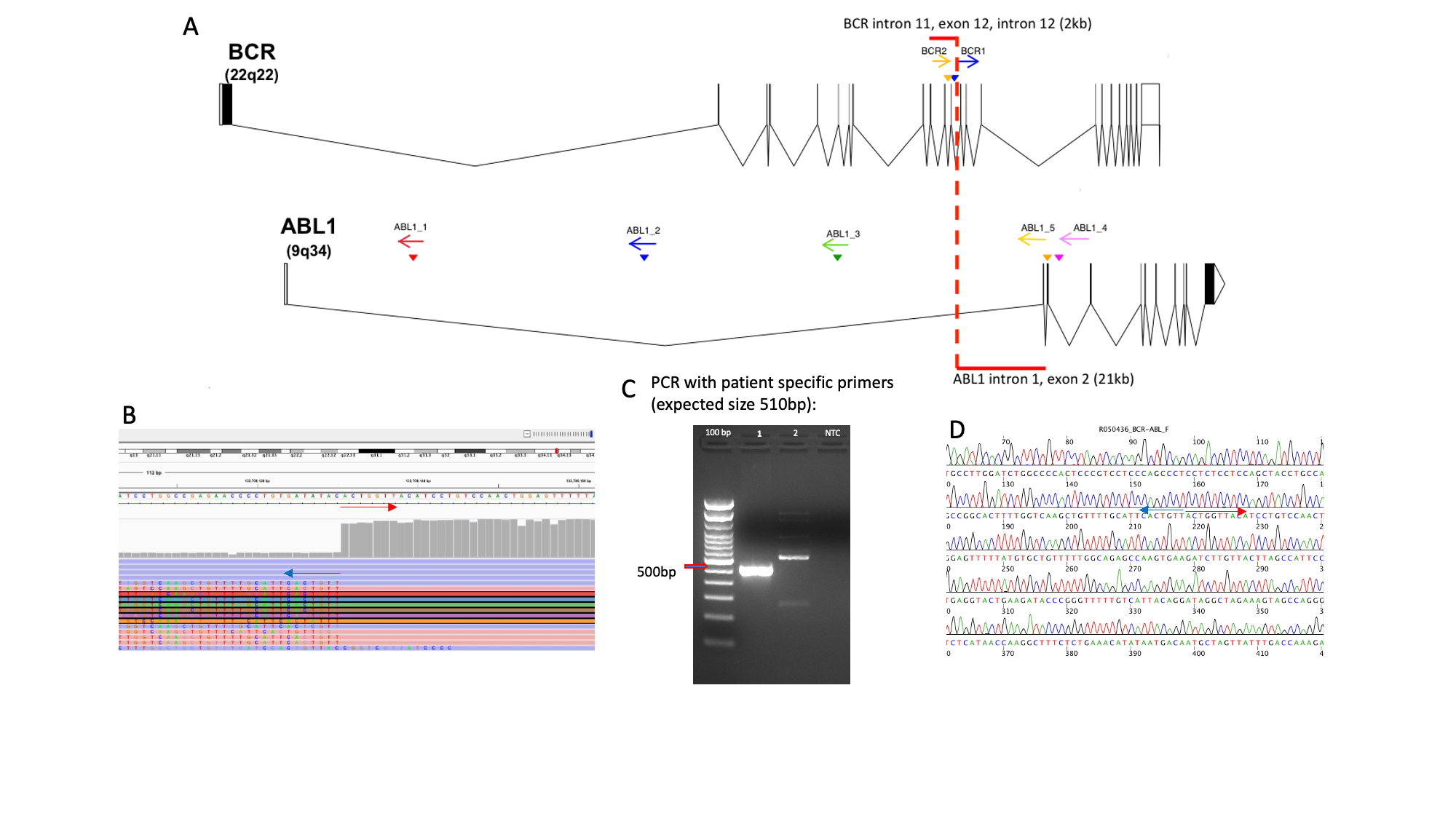

Supplement: S1 Fig — A) Schematic representation of the breakpoint captured with our amplification-free enrichment protocol and long-read sequencing. Reads capturing the breakpoint are up to approximately 23Kb long and represented in the graph by the red line: 2KB in BCR, chromosome 22, and 21KB in ABL1, chromosome9. B) Nanopore sequence IGV visualization showing the alignment of multiple sequences with ABL1 (red arrow). The fragment marked by the blue arrow does not align with ABL1 and corresponds to BCR. C) Agarose gel electrophoresis (2% agarose) of PCR amplified product using patient specific primers designed after breakpoint detection. The gel shows the patient sample in lane 1 and a BCR-AB1 cell line (KCL22) in lane 2. D) Confirmation on the patient specific breakpoint by Sanger sequencing. (TIFF) [file pgph.0002267.s002.tiff]
