# Supplementary material for: Rapid detection of myeloid neoplasm fusions using single-molecule long-read sequencing
Source: PLOS Glob Public Health. 2023 Sep 12;3(9):e0002267. doi: 10.1371/journal.pgph.0002267 (PMC10497132; doi:10.1371/journal.pgph.0002267)
Supplement: Attachment — Submitted filename: Response_cover20230608.pdf [file pgph.0002267.s004.pdf]

SCHOOL OF ENGINEERING & TECHNOLOGY
UNIVERSITY of WASHINGTON | TACOMA

February 21, 2023

Hugh Cowley
Staff Editor
PLOS Global Public Health

Re: **REVISED** PGPH-D-22-01725: *Rapid detection of myeloid neoplasm fusions using single-molecule long-read sequencing* submitted to PLOS Global Public Health

Dear Editors,
Thank you for your prompt response. Here are our point-by-point responses (shown in *italic blue*) to your requests:

Reviewer #2: The effort of the authors to meticulously revise the manuscript is appreciated.

I wish to further clarify the following 2 points:

1. Patient CML3: this patient is diagnosed as long standing p190 CML but now the disease is MDS/MPN and the BCR::ABL1 fusion transcript is at a low level. Is the patient on TKI treatment and there is clonal escape to another genetic driver, or is this a therapy-related secondary disease?
Thank you for your question for clarification of patient CML3. Unfortunately, thorough explanation of this patient may result in a case report as this patient had a very complex complete history. The patient was being treated with a TKI. Prior to the testing sample used in this study, the patient had a long-standing history of p190 CML. At the timepoint of nanopore testing, the peripheral blood sample which was available showed very low levels of BCR-ABL1, which was tested by both qualitative PCR and quantitative PCR. Only the qualitative PCR was positive, which meant the BCR-ABL1 level in the PB was below 0.01%. In subsequent timepoints (6 and 8 months later) where marrow biopsies were conducted for clinical purposes, but archival research specimens were not collected - this patient developed 3 abnormal populations including an expanded immature monocyte population, a T lymphoblast population, and an immature myeloid population and it was postulated that this could represent either a CML in blast crisis with an unusual bilinagel acute leukemia or a CML with evolved T-ALL blast crisis and a secondary MDS/MPN CMML. We did not include more data on this patient since the sample was archived nearly 20 years ago and further clinical testing was not conducted at that time, furthermore we did not conduct single cell sequencing as it was not a focus of this study.

2. Patient APL6: please clarify the meaning of 22% blasts in a < 5% marrow.

The overall marrow only has less than 5% marrow cellularity. Typically, that means that 22% blasts seen by flow cytometry in such a low cellular marrow would be much lower likely closer to less than 5% actual blasts nuclei since most of the cellularity would comprise primarily of lymphocytes, plasma cells, stromal cells and other vascular nuclei thus making bulk sequencing which considers all nuclei in the sample.

Reviewer #3: It is a well-written paper to describe a long-read sequencing DNA assay designed with CRISPR guides to select and enrich recurrent leukemia fusion genes, that does not need a priori knowledge of the abnormality present. The authors have developed an amplification-free CRISPR-Cas9 targeted enrichment sequencing protocol using Nanopore MinION flow cell and Flongles to detect fusions relevant to the diagnosis and classification of CML and AMLs.

One of the questions that I believe arises after reading the paper is: is there a specific tumor fraction above which this technology works? can a dilution series experiment be performed using a high tumor fraction sample or cell line to understand how the technology performs as a function of tumor fraction?

Thank you for this comment. In this proof-of-concept study, we have performed testing on a limited set of challenging samples to test the limits of the system. If you refer to Table 1 – CML 1, 4 and 5 were detected and they had respective disease burdens of 2%, 3% and 3%, whereas CML3 with disease burden below 0.01% was not detected. Similarly, APL1 and APL6 with low blast burden was not detected. In future experiments, we intend to perform more extensive validation on serial dilutions of multiple cell lines and patient samples to test and obtain a true LOD for each of the targets in our panel.

Minor comments:
The font sizes maybe different before and after line 54 (lines 56-64)
higher quality images would make the figures easier to read. it'll be great if this can be updated
This is corrected in the revised manuscript.
In this revision, we have submitted higher resolution images, now at 19.3MB file size.

Reviewer #4: I must admit this describes a well thought experiment. The manuscript is well detailed. I however have a couple of queries from the different section of the manuscript.
Methods
Line 51: The manuscript mentions that the protocol described takes could be performed in only 8 hours. I however realize that the sequencing run on the nanopore device oftentimes run overnight (from your response to another reviewer) and from my experience with nanopore know most run requires a minimum of about 12 hours to attain good read depth.

Thank you for sharing your experiences, our lab has also allowed nanopores to run overnight in the past. However, we began to ask what if we stopped it sooner and at what point can we generate enough data to call a fusion – thus this was how these experiments began. We looked at timed packets of data and at what point there were enough total reads and enough fusion positive reads to confirm the sample has a fusion.

Line 165: The word "Does not require previous knowledge of target" is misleading since the CRISPR guide system used is specific to concentrate known (specific) fusion regions.
We could not find this phrase in line 165, however this is seen at line 451 and we have corrected it to say : "require previous knowledge of only one of two target genes as guides can capture an unknown partner gene".

Line 197: Sanger sequencing was used to confirm the BCR-ABL breakpoint, what method was used to verify the other identified breakpoints?
Karyotype and FISH, Published cell line data.
Lines 196- 199 We have updated this section to be clearer and relabeled as Orthogonal confirmation of Fusions and Breakpoints.

The re-written paragraph is as follows PMID are included for ease of review:

> Patient specific *BCR::ABL1* breakpoints were confirmed by performing PCR and sanger sequencing in 2 cases. Primers were designed using Primer3 v. 0.4.0.(22). 100 ng of DNA were amplified, the PCR product was run on a 2% agarose gel, and Sanger sequenced to confirm the genomic breakpoint. All other patients were confirmed with clinical karyotype and fluorescent in-situ hybridization data. BCR-ABL cell lines breakpoints were confirmed by published data (PMID 19215797)"

The possibility of multiplexing sequencing run with nanopore is another advantage of the described assay which I think should be highlighted.
Was this experiment repeated to ascertain the precision of the described assay/protocol?
In this proof-of-concept study, we have performed testing on a limited set of samples. While some cell lines have been repeatedly tested (K562, NB4), the patient samples are precious and at this point we have not performed reproducibility testing. These are however planned for future testing in validation studies.

Sincerely,

Ka Yee Yeung, Ph.D.
Professor
University of Washington, School of Engineering and Technology
