# Supplementary material for: Rapid detection of myeloid neoplasm fusions using single-molecule long-read sequencing
Source: PLOS Glob Public Health. 2023 Sep 12;3(9):e0002267. doi: 10.1371/journal.pgph.0002267 (PMC10497132; doi:10.1371/journal.pgph.0002267)
Supplement: Attachment — Submitted filename: Response_ky2_response.docx [file pgph.0002267.s003.docx]

**REVISED** PGPH-D-22-01725: *Rapid detection of myeloid neoplasm fusions using single-molecule long-read sequencing* submitted to PLOS Global Public Health

We appreciate the reviewers’ thoughtful comments and have made major modifications to the manuscript. In addition, we offer the additional comments suggesting certain points below.

**Reviewer #1:** Excellent and well-designed study.

I would like to kindly inquire about the following:

- Why wasn't RNUX1-RUNX1T1 tested?

We thank the reviewer for this comment. Probes were designed to target RUNX1::RUNX1T1 but have not been fully optimized by the time of this submission. Our first diseases to focus on included APL, CML and AML with fusions that are difficult to detect by current standard of care FISH or PCR methods such as KMT2A fusions.

- What is the claimed minimum %read alignment adopted for "excellent" quality?

Per the manufacture and the current standards adapted by the nanopore community there is no established QC parameter for what minimum % read alignment should be considered excellent. The reviewer is very insightful with this question, and we have also noted in our data that % of reads aligned does not correlate with PHRED scores and as such have tracked both parameters. PHRED scores ranged from 8.95 to 12.94, and % reads aligned ranged from 72.08% to 96.29%. (see revised table 2) One example showed 72.08% reads aligned with a PHRED score of 11.15; however, the important parameter is the confirmation of the orthogonal clinical data despite a low % read alignment. This is an astute observation and requires more study to understand why we have this observation. (Line 314-316, Lines 344-346)

In the experiments described in this manuscript we have trimmed reads and have not filtered or discarded reads with low PHRED scores prior to alignment.

- Were the discrepant cases ever retested using RNA instead of DNA as a starting genomic material?

Thank you for your comment. The CRISPR Cas9 assay is designed to target DNA, hence guides do not necessarily target exon sequences. We could not use this same assay on RNA.

- Kindly check grammar mistakes throughout the manuscript

Thank you for this comment – we have performed a thorough grammar edit. We have submitted two versions of the manuscript, one with all tracked changes and one clean version to reflect all grammatical edits and changes that were made.

**Reviewer #2:** Sala-Torra et al applied PCR-free CRISPR-Cas9 targeted DNA nanopore sequencing to detect gene fusions from 6 cell lines and 14 clinical samples. This reviewer has the following reservations regarding study design, Page 4 of 5 methods, results and conclusions.

1. Study design

- (lines 150-154) Guide RNA are designed in both forward and reverse directions of each entire gene? Does "MYH11- CBFB" mean that the canonical driver fusion CBFB-MYH11 is also covered? Are PML-RARA bcr1/bcr2/bcr3 variants all covered? Is the BCR-ABL1 p210 covered but not p190?

Thank you. Guides are designed to cover the fusion of interest (in the appropriate direction for each gene to capture the relevant fusion). Guides for inv(16) are designed on the positive strand of CBFB cutting upstream of exon 4, and in the positive strand of MYH11, downstream of exon 33 or 34 (NM_022844.2 or NM_002474.3, MYH11 gene being in the negative strand) so fusion reads span the junction CBFB exon 4- MYH11 exon 33 or 34. Guides are design to target both partners in a fusion gene PML-RARA and BCR-ABL1. See next question for BCR-AVBL1 p210/p190 answer.

- (Table 1) Sample "CML3" has p190 isoform but the assay is designed to detect p210 (line 153). All 3 BCR-ABL1 cell lines have p210 isoform as well (K562:b3a2, KU812:b3a2, KCL22:b2a2). False negative detection can be attributed to assay design instead of "low disease burden" (line 298).

While BCR-ABL1 was optimized for p210 (see figure below), the CRISPR guides which sit on the ABL1 gene could be able to detect and capture both isoforms including p190.

- (lines 335-339) Flongle and MinION output comparison (data output yield, read length, phred score) are not based on parallel run using the same input DNA and sequencing library. Input DNA quality and sequencing library preparation batch effect could be confounding factors.

Thank you. The reviewer is correct in that the same library was not tested on a flow cell and a Flongle. These are meant to be single use experiments and will need to have two flow cells (MinION and Flongle) run in parallel with a double library prep, while our system is set up for single device to be run at a time. We thank the reviewer for this suggestion and have planned to conduct these quality control experiments in the future. The goal of this manuscript is a report on our proof of concept and our latest success.

- If CML is suspected, will RT-PCR or GeneXpert cartridge be cheaper and quicker alternative to detect BCR-ABL1 fusion for low/middle income countries?

Yes only if they have the parent instrument, if the diagnosis of CML or APL is known, and if no other fusion genes are required to be ruled out. This is difficult in the global oncology setting as not only are there shortages of molecular laboratories and pathologists there are also a shortage of hematologist/oncologists. Identification of specific key targetable fusion genes are intended to allow for life saving treatments to be given even when resources are low.

2. Methods

- (line 168) Is a new flow cell is used for each sample? Same run time of 72 hours? Which Guppy basecaller version and basecalling model?

Yes, a new flow cell was used for every experiment. Active sequencing times of the nanopore device varied and were often left overnight, however during the post run data analysis we could model the data analysis based on shorter run times. A new flow cell was used for every sample. Basecalling was performed using Guppy version 6.4.6 using the r9.4.1 hac model. We have updated the manuscript lines 345-350 with the following passage:

“We have recently developed a Bwb workflow(19) to support the processing of Nanopore data that includes the use of base callers Guppy(25) version 6.4.6 using the r9.4.1 hac model, alignment using minimap2 and visualization of results using the Integrative Genomics Viewer (IGV) and GRCh37 hg19 as the reference genome. QC data was generated with PycoQC.(26) Minimap2(27) was used as the aligner and variant caller. “

- Is the BFF source available for readers to implement in their own lab?

A provisional patent has been filed on this tool and we intend to make BFF available upon request through academic licenses, collaborations, and other academic arrangements.

3. Results

- (Table 2) Results table and paragraph are difficult to read and interpret. For each sample (cell line, clinical sample), what is the expected result, true positive / false negative fusion detected, absolute number of reads, and how many false positive fusion calls >= 3 reads. Enrichment ratio is a good sample-level QC metric. However, for a given fusion, e.g. BCR-ABL1, absolute number of reads (fusion spanning reads for true positive, average read count for false negative) is needed for interpretation. In fact Table 2 is incompletely shown in the PDF file and the right hand side portion is missing.

Thank you for this comment – we have reorganized Table 2 and split it into two tables: a new Table 2 that is focused on the characteristics of the run, and a new Table 3 which focuses of the enrichment and comparison of the performance of 2 fusion detection tools for easier comprehension and readability. We agree with the reviewer and thought this strategy would help audiences better interpret the data and make the article easier to read.

- (line 323) Sample bioinformatic analysis time 188 minutes is inconsistent with discussion (line 358: 4.5 minutes)

188 minutes is the total sequencing and processing time to detect 3 fusion reads, which includes both the real-time sequencing reaction and the data analysis.

The 4.5 minutes is the amount of computation time to process this amount of data through the bioinformatics pipeline.

We have updated lines 598 to clarify that this is for sequencing and fusion to detect a minimum of 3 fusion reads, and in line 654 we indicate that ~4.5 minutes is only for “base calling, alignment, and fusion detection”.

- Fusion genes should be consistently presented using conventional names (MLL-AF4) or latest nomenclature

(KMT2A-AFF1) (line 293) but not a mix (KMT2A-AF4) (line 42). More examples include KMT2A-SEPTIN9 or MLL-SEPT9 but not KMT2A-SEPT9 (Line 313)

Thank you for bringing these inconsistencies up to our attention. All gene fusions have been updated to the newest nomenclature rules BCR::ABL1, PML::RARA, KMT2A::AFF1, KMT2A::MLLT3, KMT2A::SEPTIN9, and CBFB::MYH11.

- (Table 2) Sample "CML6" what is "in" BFF fusion read count?

This has been corrected to 106

- (Table 2 and line 168) Flongle is also flow cell. Therefore, "flowcell" is ambiguous and confusing. Flongle versus MinION flowcell may help to differentiate.

This has been corrected. We have implemented your suggestion and used MinION flowcell instead of flowcell throughout the manuscript for clarity.

4. Conclusion

- (line 434) Does not require previous knowledge of target? but the presented assay is targeted sequencing not whole genome sequencing

The purpose of this assay was designed for AML and CML for which there are known recurrent fusion targets and when detected helps identify patients that can benefit from specific treatments. When we indicate “not require previous knowledge of target”, we mean in any patient with a high white count and leukemia is suspected, this assay will be able to not only identify if there is AML or CML but also be able to identify a targetable fusion gene.

- (line 434) TAT: Is sample extraction and library preparation and bioinformatics time is included?

Yes, TAT indicates from sample to report of fusions.

- (line 436) Since data is not shown for genomic breakpoint sequence nor are the primers available for review, it Page 5 of 5 remains to be shown how one can precisely map translocation genomic breakpoint by this assay.

Thank you for pointing this out, we have included a figure below demonstrating how we orthogonally confirmed precise fusion breakpoints and added to table 3 the precise fusion breakpoints coordinates.

We have added the following passages to added clarity in response to this point:

In the Methods Section, line 240-241

“BFF will identify the precise breakpoint in each read and also give a count of the number of breakpoints found nearby in all reads.”

In the Results Section, line 330-333

” BFF provides the precise breakpoint in each read in addition to the number of breakpoints found nearby (see table 3). LongGF only provides the precise breakpoints when there is enough supporting reads.”

We have also added an extra column in Table 3 showing the precise break points that are provide by BFF.

We further offer the following figure in which orthogonal confirmation of the specific breakpoints was conducted for select patient cases via PCR (see below figure) and fusion breakpoints of cell lines were compared to published data.

We have not included this data as it we thought it would be bulky and would detract from the main point of the paper. If the reviewer or editor feels this is helpful as a supplemental figure, we are happy to include with the manuscript.

   

Description of the figure: Genomic BCR-ABL1 breakpoint identified in a primary specimen by long-read sequencing. A) Schematic representation the breakpoint captured with our amplification-free enrichment protocol and long-read sequencing. Reads capturing the breakpoint are up to approximately 23Kb long and represented in the graph by the red line: 2KB in BCR, chromosome 22, and 21KB in ABL1, chromosome9. B) Nanopore sequence IGV visualization showing the alignment of multiple sequences with ABL1 (red arrow). The fragment marked by the blue arrow does not align with ABL1 and corresponds to BCR. C) Agarose gel electrophoresis (2% agarose) of PCR amplified product using patient specific primers designed after breakpoint detection. The gel shows the patient sample in lane 1 and a BCR-AB1 cell line (KCL22) in lane 2. D) Confirmation on the patient specific breakpoint by Sanger sequencing.
